# Breast Cancer Survival Rates in Costa Rican Social Security Fund Patients: A 5-Year Analysis

**DOI:** 10.4236/jss.2023.119018

**Published:** 2023-09-18

**Authors:** Amada Aparicio, Percy Guzman, Esmeralda Ramirez-Pena, Melvin Morera Salas, Michael Halpern

**Affiliations:** 1 Costa Rica Social Security Fund, San Jose, Costa Rica; 2 Cancer Prevention Fellowship Program (CPFP), Division of Cancer Prevention (DCP), National Cancer Institute (NCI), Maryland, USA; 3 Health Assessment Research Branch (HARB), Health Delivery Research Program (HDRP), Division of Cancer Control and Population Sciences (DCCPS), Maryland, USA; 4 Surveillance Research Program (SRP), Division of Cancer Control and Population Sciences (DCCPS), National Cancer Institute (NCI) Maryland, USA

**Keywords:** Breast Cancer, Survival, Risk Factors, Treatment

## Abstract

**Background:**

Breast cancer is a significant cause of mortality among women worldwide. While there has been increasing focus on breast cancer screening in countries of Central American, limited recent information is available providing national survival rates. This study aimed to determine the 5-year survival rate for women with breast cancer who received treatment through the Costa Rican Caja Costarricense del Seguro Social (CCSS) between 2008 and 2012.

**Methods:**

A retrospective cohort study was conducted of women with breast cancer who received treatment through the CCSS between 2008 and 2012. The Kaplan-Meier (KP) method was used to calculate survival rates, and univariate and multivariate Cox regression analyses were conducted to identify factors associated with survival.

**Results:**

The KP method showed a gradual decline in survival over time, declining from 94.4% at 12 months to 76.3% at 5 years. The 5-year survival rates were highest in early-stage (stage I and IIA) and lowest in stage IV; five-year mortality rate among women diagnosed with stage IIIB cancer was four times higher than for those with stage IIB cancer and half that of women diagnosed with metastatic disease. Patients who underwent conservative or radical surgery had decreased risks of mortality compared to those who did not receive surgery. Multivariate analysis identified advanced stage, older age, multifocal tumors, poorly differentiated tumors, triple-negative tumors, and the absence of surgery as significant risk factors for mortality.

**Conclusion:**

The 5-year survival rate for women with breast cancer who received treatment through the CCSS between 2008 and 2012 is encouraging, but improvement is needed in early diagnosis and treatment, particularly for women with advanced stages of breast cancer. These findings highlight the need for continued efforts to improve breast cancer detection and treatment in Costa Rica.

## 1. Introduction

Cancer is a major health challenge worldwide, with over 70% of cancer deaths occurring in low- and middle-income countries. Early detection can prevent at least one third of cancer cases that occur annually worldwide, according to the World Health Organization (World Health Organization, 2021).

Breast cancer is the most frequently diagnosed cancer among women world-wide (International Agency for Research on Cancer, 2021) and is the leading cause of cancer death among women in Costa Rica (Caja Costarricense de Seguro Social, 2020). Previous estimates reported an increase in incidence of 6.5% from 2000 to 2020, reaching rates of 11.5 per 100,000 population (International Agency for Research on Cancer, 2018). Recent efforts in Costa Rica have focused on improved breast cancer screening and increased access to high-quality treatment among women diagnosed with breast cancer (UT Southwestern Medical Center, 2021). However, limited information is available to assess the effects of these initiatives on survival among women diagnosed with breast cancer at a national level. This analysis was conducted to examine survival rates and factors associated with survival among women diagnosed with breast cancer in Costa Rica using national data from the Costa Rican Social Security Fund (Caja Costarricense del Seguro Social, CCSS).

## 2. Methodology

### 2.1. Population and Data Collection

The study population consisted of all women aged 16 years or older who were registered in the National Tumor Registry (NTR) database and diagnosed with invasive breast cancer between January 1st, 2008, and December 31st, 2012. A total of 4775 women diagnosed with breast cancer within this period were initially identified, but several groups were excluded from the cohort, including 18% (n = 864) diagnosed in private establishments, 2% (n = 75) with unknown place of diagnosis, 6% (n = 285) treated in health centers that diagnosed less than 50 cases, and 8.2% (n = 392) without a file or clinical history. The resulting cohort of 3,160 patients was followed for up to 60 months.

Data collection was performed by CCSS getting information like, identifiers (name, identity, and all the medical history, including diagnostic, treatment and patients’ follow up) extrapolated with the NTR information, which controls the quality, validity, and consistency of information. Dates of death from 2008 to 2017 were obtained from several sources, including the NTR, medical centers, digital records, and death certificates, and were validated in the database of the Supreme Electoral Tribunal (SET), the Costa Rican government organization where all births and deaths are registered. Cases where hospital files were not located were included in the study database with only the information from the NTR and the SET, including the date of birth, date of pathological diagnosis, and date of death.

### 2.2. Outcome Measures

The primary outcome measure was 5-year survival of women diagnosed with breast cancer between 2008 and 2012. Mortality was calculated as the proportion of deaths among the cohort of patients followed for up to 60 months. The dates of death from 2008 to 2017 were obtained from several sources, including the NTR, medical centers, digital records, and death certificates, and were validated in the database of the Supreme Electoral Tribunal (SET), the Costa Rican government organization where all births and deaths are registered. Cases where hospital files were not located were included in the study database with only the information from the NTR and the SET, including the date of birth, date of pathological diagnosis, and date of death.

### 2.3. Data Analysis

Descriptive statistics were used to summarize the demographic and clinical characteristics of the study population, including age, stage at diagnosis, and treatment modality. Continuous variables were summarized as means with standard deviations and medians with ranges, while categorical variables were summarized as relative frequencies, ratios, and 95% confidence intervals (CI). In addition to data from the NTR, other data were collected using a standardized data collection form and entered into an Excel 97–2003 format using a standardized code.

Descriptive statistical analysis was performed using SPSS and STATA version 17 programs. The Kaplan-Meier method was used to estimate the survival at several time points, and survival curves were compared using the log-rank test. Multivariate Cox regression analysis was performed to identify independent predictors of survival, including age, stage at diagnosis, and treatment modality. The statistical significance level was set at 0.05.

Ethical considerations were observed throughout the study, and patient confidentiality was maintained at all times. The study protocol was evaluated and approved by the Central Scientific Ethics Committee, which is part of the Center for Strategic Development and Information on Health and Social Security of Costa Rica. The study was registered with the National Council for Health Research under the code R015-SABI-00074 (Central Scientific Ethics Committee, Costa Rica Social Security Fund, 2008–2012).

## 3. Results

### 3.1. Observation Time and Mortality Rates

The cohort of women was followed with a minimum time of 0.03 months following diagnosis and a maximum of 60 months. The interquartile range was (11.2 – 40.7), and the median follow-up time was 29.5 months. The mortality rate for the period 2008–2012 was 4.6 deaths from invasive breast cancer per 1000 women-months of follow-up (95% CI: 4.1 – 5.1).

### 3.2. Survival Rate

**Figure 1.**
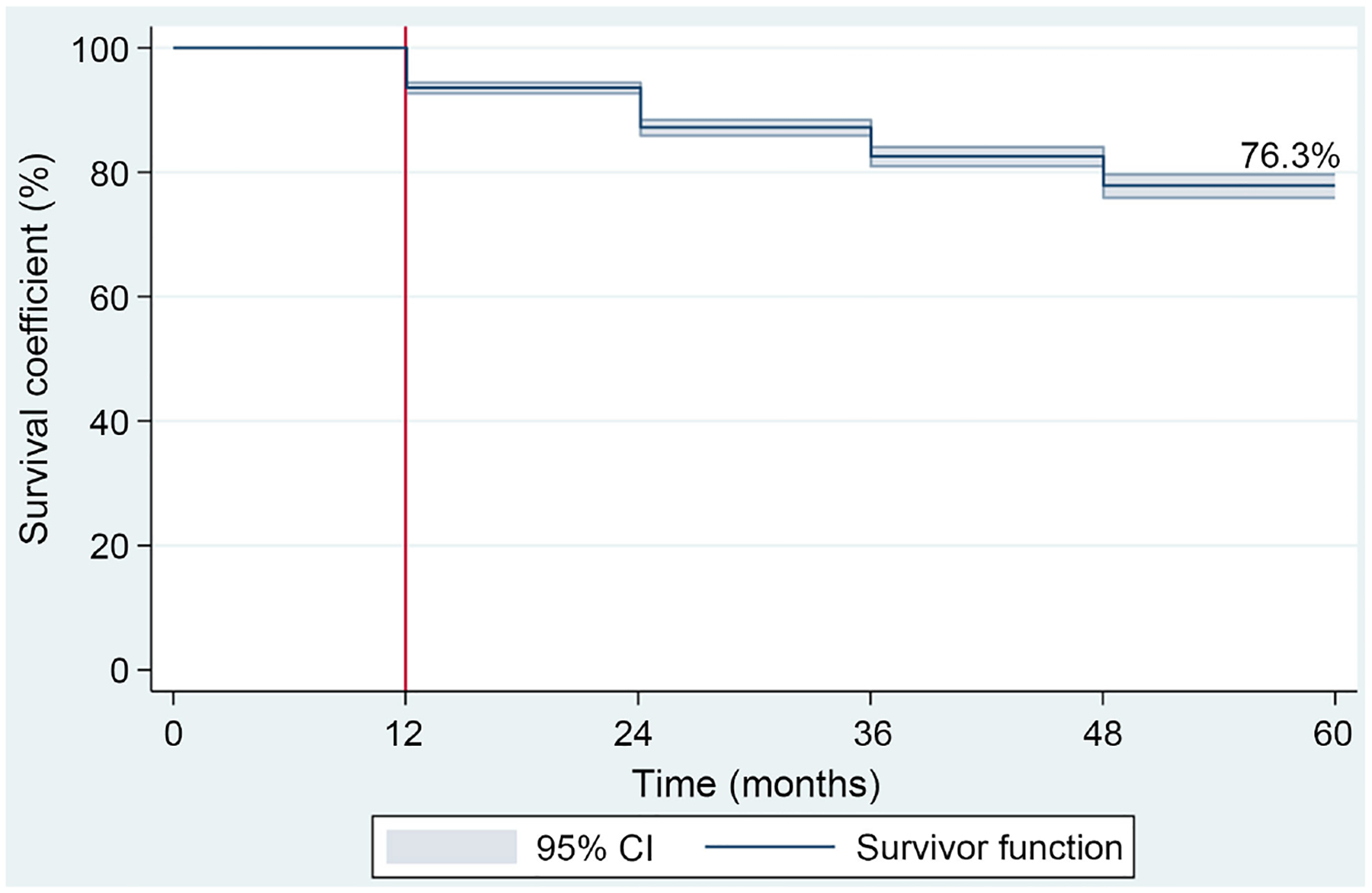
Kaplan-Meier method, overall survival.

**Figure 2.**
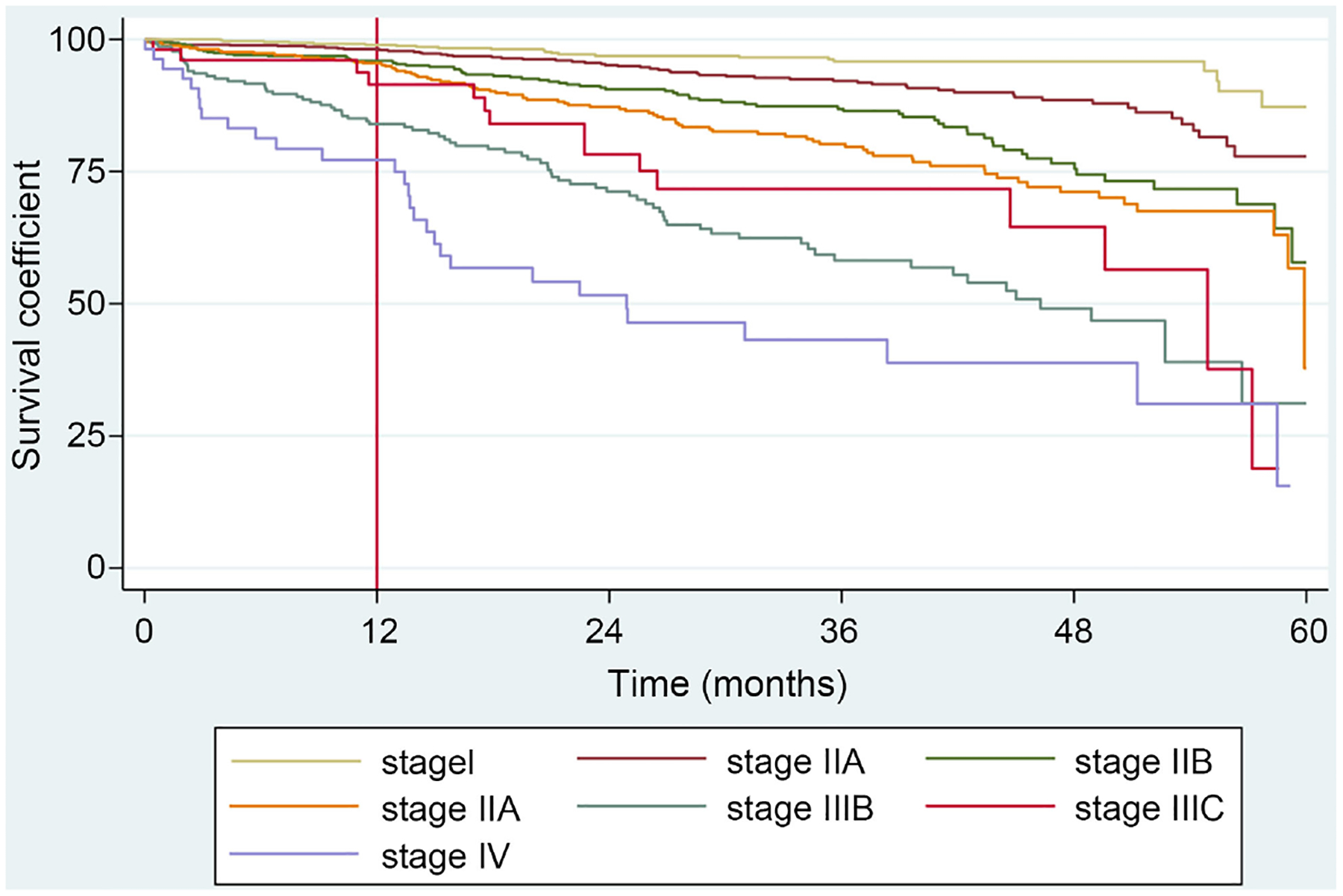
Kaplan-Meier method, by stage (months).

The overall five-year survival rate for all women included in the study was 76.3% (Figure 1). The five-year survival rates for each clinical stage were: stage I (98.4%), stage IIA (96.5%), stage IIB (92.6%), stage IIIA (96.5%), stage IIIB (73.7%), stage IIIC (78.9%), and stage IV (61.1%) (Table 1 and Figure 2).

### 3.3. Survival Analysis

As expected, the Kaplan-Meier curve showed a decline in survival over time (Figure 1). Survival rates were 94.4% (95% CI = 93.5 – 95.2) at 12 months, 89.0% (95% CI = 87.7 – 90.2) at 24 months, 85.3% (95% CI = 83.7 – 86.8) at 36 months, 81.3% (95% CI = 79.2 – 83.2) at 48 months, and 76.3% (95% CI = 72.6 – 79.6) at 60 months.

The five-year mortality rates increased with more advanced stage at diagnosis. Women diagnosed with stage IIIB breast cancer had a five-year mortality rate four times higher than those diagnosed with stage IIB and two times lower than those diagnosed with metastatic disease. The highest five-year mortality rates were observed in patients with stage IIIB (25.89%), IIIC (21.15%), and stage IV (38.89%) breast cancer. In contrast, patients with stage I (1.46%), IIA (3.08%), IIB (6.87%), and IIIA (9.93%) breast cancer had five-year mortality rates below 10%. These results are summarized in Table 2.

### 3.4. Breast Cancer Mortality and Risk Factors

Univariate and multivariate analyses were conducted to identify risk factors for breast cancer survival. In the univariate analysis, increased five-year mortality rates were observed for patients with stage III disease at diagnosis (hazard ratio (HR): 4.53; 95% confidence interval (CI): 3.38 – 6.07) or metastatic (stage IV) disease at diagnosis (HR: 14.50; 95% CI: 8.92 – 23.54) breast cancer (compared with those with earlier stages, stage I/II); multi-centric (HR: 4.17; 95% CI: 0.83 – 1.50) and multi-focal (HR: 4.87; 95% CI: 2.61 – 9.08) tumors (vs. breast cancer limited to the upper outer quadrant); moderately (HR: 2.33; 95% CI: 1.43 – 3.78) and poorly (HR: 4.32; 95% CI: 2.65 – 7.06) differentiated tumors (vs. well differentiated); and HER2-positive (HR: 2.45; 95% CI: 1.61 – 3.74), and triple-negative (HR: 2.93; 95% CI: 2.13 – 4.04) cancers (vs. hormone-receptor positive/HER2-negative or luminal A cancers). Patients who underwent conservative (HR: 0.04; 95% CI: 0.03 – 0.05) or radical (HR: 0.08; 95% CI: 0.06 – 0.10) surgery had decreased risks of five-year mortality compared to those who did not receive surgery (see Table 3). However, this may reflect stage at diagnosis and/or the presence of other substantial comorbidities that could decrease the likelihood of surgical treatment and independently increase mortality risk.

In multivariate Cox (proportional hazards) analysis, advanced stage at diagnosis (HR: 2.03; 95% CI: 1.45 – 2.84) was a significant risk factor for five-year mortality compared to earlier stages. However, there was no statistically significant difference in mortality risk observed between patients with metastatic breast cancer and those with earlier stage disease at diagnosis (HR = 1.47; 95% CI: 0.83 – 2.58; *p* = 0.186). Patients older than 65 years at diagnosis (HR: 1.54; 95% CI: 1.02 – 2.32) had a significantly elevated mortality risk compared to those younger than 46 years. Patients with multifocal tumors (HR: 2.88; 95% CI: 1.52 – 5.45) had a significantly increased risk of mortality compared to those with tumor limited to the Upper Outer Quadrant (UOQ). Patients with moderately (HR: 2.21; 95% CI: 1.33 – 3.65) and poorly (HR: 2.57; 95% CI: 1.53 – 4.33) differentiated tumors were associated with a higher risk of five-year mortality than were those with well-differentiated tumors, while triple-negative tumors (HR: 1.72; 95% CI: 1.18 – 2.53) were associated with a greater risk than were luminal A tumors. Surgery, whether conservative (HR: 0.48; 95% CI: 0.29 – 0.78) or radical (HR: 0.52; 95% CI: 0.35 – 0.79), was associated with decreased risks of mortality compared to those who did not receive surgery (Table 4).

## 4. Discussion

In conclusion, this study has reported multiple factors associated with five-year mortality among women diagnosed with breast cancer in Costa Rica, contributing valuable insights into the national-level estimates of breast cancer mortality rates (Fayanju et al., 2018). Our findings align with previous research, emphasizing the importance of timely and appropriate screening and treatment for breast cancer, especially for patients with advanced-stage disease, older age, and poorly differentiated tumors, as these factors were associated with increased breast cancer mortality (Chen et al., 2017; Wang et al., 2019; Fayanju et al., 2018).

However, it is essential to recognize the potential impact of lead-time bias on our survival estimates, particularly at early stages of breast cancer. Lead-time bias occurs when the early detection of cancer through screening leads to the appearance of prolonged survival, not necessarily due to improved treatment outcomes but because of earlier diagnosis (Chen et al., 2017). While our study provides valuable information on breast cancer survival rates in Costa Rica and identifies significant risk factors associated with increased mortality, the possibility of lead-time bias should be considered when interpreting survival estimates, especially for cases detected through screening programs.

Moreover, our study highlights the critical role of both conservative and radical surgery in improving survival rates among breast cancer patients, consistent with previous research (Houssami et al., 2014; Lund et al., 2009). Additionally, the identification of patients with triple-negative breast cancer is crucial for directing targeted treatment approaches and improving survival rates (Ensenyat-Mendez et al., 2021).

These findings underscore the importance of national estimates in developing targeted strategies to decrease breast cancer mortality rates in Costa Rica. Interventions to improve access to screening and diagnosis, enhance treatment options and quality, and provide supportive care can be prioritized based on the risk factors identified in our study. The results emphasize the need for timely and appropriate treatment, particularly for women with advanced age, poorly differentiated tumors, triple-negative disease, or metastatic disease.

In summary, our study adds to the growing body of literature on breast cancer survival and risk factors, providing key information on breast cancer survival in Costa Rica, a middle-income country with limited published data in this area (Fayanju et al., 2018). The identification of these risk factors can help clinicians make more informed decisions about treatment options, and policymakers identify priority areas to improve patient outcomes. The findings underscore the importance of early detection and treatment of breast cancer and emphasize the need for targeted interventions to reduce breast cancer mortality rates in Costa Rica while considering the potential impact of lead-time bias on our estimates (Chen et al., 2017).

## Figures and Tables

**Table 1.** Five-year survival by breast cancer stages.

Variable	Cumulative Survival (%)	
Clinical Stage		
I	676/687	(98.4)
IIA	815/845	(96.5)
IIB	526/568	(92.6)
IIIA	389/143	(96.5)
IIIB	165/224	(73.7)
IIIC	41/52	(78.9)
IV	33/54	(61.1)
Unknown	126/297	(42.4)
Grouped Stages		
Early	2.017/2.100	(96.1)
Advanced	595/709	(83.9)
Metastatic	33/54	(61.1)

**Table 2.** Five-year mortality from breast cancer according to clinical stage.

Stage	Cumulative lethality		Case fatality rate		
	(%)	*p*	FU*	Rates (CI 95%)	*p*
I	10/687 (1.46)		19.016.63	0.53 (0.28 – 0.98)	
IIA	26/845 (3.08)		22.796.56	1.14 (0.78 – 1.68)	
IIB	39/568 (6.87)		15.168.40	2.57 (1.89 – 3.52)	
IIIA	43/433 (9.93)	X^2^: 811.83	12.178.86	3.5 (2.62 – 4.76)	X^2^: 980.29
IIIB	58/224 (25.89)	*p*: 0.000	5.230.94	11.09 (8.57 – 14.34)	*p*: 0.000
IIIC	11/52 (21.15)		1.350.14	8.15 (4.51 – 14.71)	
IV	21/54 (38.89)		1.099.49	19.10 (12.45 – 29.29)	
Unknown	170/297 (57.24)		5.876.23	28.93 (24.89 – 33.62)	

* FU: Follow-up time in month.

**Table 3.** Univariate analysis according to Cox Regression.

Variable	HR (CI 95%)	*P*
Grouped Stages		
Early	**Reference**	
Advanced	4.53 (3.38 – 6.07)	0.000
Metastatic	14.50 (8.92 – 23.54)	0.000
Age grouped		
<46 years	**Reference**	
46 – 54	0.53 (0.38 – 0.75)	0.000
55 – 64	0.70 (0.51 – 0.97)	0.034
≥65	1.12 (0.83 – 1.50)	0.463
Localization		
UOQ	**Reference**	
LOQ	1.09 (0.67 – 1.78)	0.738
UIQ	1.07 (0.71 – 1.61)	0.734
UIQ	0.80 (0.43 – 1.49)	0.486
Retro areolar	1.57 (0.92 – 2.71)	0.101
Multifocal	4.87 (2.61 – 9.08)	0.000
Multicentric	4.17 (2.87 – 6.07)	0.000
Differentiation		
Well	**Reference**	
Moderately	2.33 (1.43 – 3.78)	0.001
Poorly	4.32 (2.65 – 7.06)	0.000
Phenotype		
Luminal A	**Reference**	
Luminal B	1.32 (0.77 – 2.25)	0.311
HER2 positive	2.45 (1.61 – 3.74)	0.000
Triple negative	2.93 (2.13 – 4.04)	0.000
Type of surgery		
No surgery	**Reference**	
Conservative	0.04 (0.03 – 0.05)	0.000
Radical	0.08 (0.06 – 0.10)	0.000

HR: hazard ratio, UOQ: Upper Outer Quadrant, LOQ: Lower Outer Quadrant, UIQ: Upper Inner Quadrant, LIQ: lower Inner Quadrant.

**Table 4.** Multivariate model of survival from breast cancer in women in the study.

Variable	HR (95% CI)	*p*
Grouped Stages		
Early	**Reference**	
Advanced	2.03 (1.45 – 2.84)	0.000
Metastatic	1.47 (0.83 – 2.58)	0.186
Age grouped		
<46 years	**Reference**	
46 – 54	0.76 (0.48 – 1.22)	0.253
55 – 64	1.02 (0.65 – 1.59)	0.946
≥65 years	1.54 (1.02 – 2.32)	0.039
Localization		
UOQ	**Reference**	
LOQ	1.10 (0.67 – 1.81)	0.707
UIQ	1.12 (0.74 – 1.70)	0.590
UIQ	0.80 (0.42 – 1.51)	0.494
Retro areolar	0.61 (0.34 – 1.10)	0.099
Multifocal	2.88 (1.52 – 5.45)	0.001
Multicentric	1.23 (0.82 – 1.85)	0.322
Differentiation		
Well	**Reference**	
Moderately	2.21 (1.33 – 3.65)	0.002
Poorly	2.57 (1.53 – 4.33)	0.000
Phenotype		
Luminal A	**Reference**	
Luminal B	1.26 (0.73 – 2.19)	0.408
HER2 positive	1.07 (0.66 – 1.73)	0.797
Triple negative	1.72 (1.18 – 2.53)	0.005
Type of surgery		
No surgery	**Reference**	
Conservative	0.48 (0.29 – 0.78)	0.003
Radical	0.52 (0.35 – 0.79)	0.002
Metastatic disease		
NO metastasis	**Reference**	
Metastasis	4.91 (3.62 – 6.65)	0.000

## Data Availability

The datasets generated during and/or analyzed during the current study are available in the Central Scientific Ethics Committee, Costa Rica Social Security Fund (2008–2012). Data on breast cancer patients reported between 2008 and 2012. National Council for Health Research registration code: R015-SABI-00074. That dataset is available through the written request directed to the Central Scientific Ethics Committee.
